# CETP (Cholesteryl Ester Transfer Protein) Inhibition With Anacetrapib Decreases Production of Lipoprotein(a) in Mildly Hypercholesterolemic Subjects

**DOI:** 10.1161/ATVBAHA.117.309549

**Published:** 2017-08-23

**Authors:** Tiffany Thomas, Haihong Zhou, Wahida Karmally, Rajasekhar Ramakrishnan, Stephen Holleran, Yang Liu, Patricia Jumes, John A. Wagner, Brian Hubbard, Stephen F. Previs, Thomas Roddy, Amy O. Johnson-Levonas, David E. Gutstein, Santica M. Marcovina, Daniel J. Rader, Henry N. Ginsberg, John S. Millar, Gissette Reyes-Soffer

**Affiliations:** From the Columbia University, New York (T.T., W.K., R.R., S.H., H.N.G., G.R.-S.); Merck & Co, Inc, Kenilworth, NJ (H.Z., Y.L., P.J., J.A.W., B.H., S.F.P., T.R., A.O.J.-L., D.E.G.); University of Washington, Seattle (S.M.M.); and University of Pennsylvania, Philadelphia (D.J.R., J.S.M.).

**Keywords:** anacetrapib, CETP, cysteine, ezetimibe, hypercholesterolemia, inhibitor, kringle, lipoprotein (a), lipoprotein metabolism, stable isotopes

## Abstract

**Objective—:**

Lp(a) [lipoprotein (a)] is composed of apoB (apolipoprotein B) and apo(a) [apolipoprotein (a)] and is an independent risk factor for cardiovascular disease and aortic stenosis. In clinical trials, anacetrapib, a CETP (cholesteryl ester transfer protein) inhibitor, causes significant reductions in plasma Lp(a) levels. We conducted an exploratory study to examine the mechanism for Lp(a) lowering by anacetrapib.

**Approach and Results—:**

We enrolled 39 participants in a fixed-sequence, double-blind study of the effects of anacetrapib on the metabolism of apoB and high-density lipoproteins. Twenty-nine patients were randomized to atorvastatin 20 mg/d, plus placebo for 4 weeks, and then atorvastatin plus anacetrapib (100 mg/d) for 8 weeks. The other 10 subjects were randomized to double placebo for 4 weeks followed by placebo plus anacetrapib for 8 weeks. We examined the mechanisms of Lp(a) lowering in a subset of 12 subjects having both Lp(a) levels >20 nmol/L and more than a 15% reduction in Lp(a) by the end of anacetrapib treatment. We performed stable isotope kinetic studies using ^2^H_3_-leucine at the end of each treatment to measure apo(a) fractional catabolic rate and production rate. Median baseline Lp(a) levels were 21.5 nmol/L (interquartile range, 9.9–108.1 nmol/L) in the complete cohort (39 subjects) and 52.9 nmol/L (interquartile range, 38.4–121.3 nmol/L) in the subset selected for kinetic studies. Anacetrapib treatment lowered Lp(a) by 34.1% (*P*≤0.001) and 39.6% in the complete and subset cohort, respectively. The decreases in Lp(a) levels were because of a 41% reduction in the apo(a) production rate, with no effects on apo(a) fractional catabolic rate.

**Conclusions—:**

Anacetrapib reduces Lp(a) levels by decreasing its production.

**Clinical Trial Registration—:**

URL: http://www.clinicaltrials.gov. Unique identifier: NCT00990808.

## Abstract 2

Supplemental Digital Content is available in the text.

**Highlights**

- In mildly dyslipidemic participants, anacetrapib lowered plasma Lp(a) [Lipoprotein (a)] levels by 34%.
- In subjects with baseline Lp(a) levels >20 nmol/L, anacetrapib lowers Lp(a) by decreasing the production of apo(a) [apolipoprotein (a)] present on mature Lp(a) particles.
- Future studies are needed to examine whether reducing Lp(a) levels will reduce the risk of cardiovascular disease.

Lp(a) [lipoprotein (a)] is a subclass of apoB (apolipoprotein B)-containing lipoprotein particles, which is typically found in the density range overlapping LDL (low-density lipoproteins) and HDL (high-density lipoproteins).^1^ The hallmark of the Lp(a) particle is apo(a) [apolipoprotein (a)], which is bound to apoB-100 by a disulfide linkage between cysteine 4326 in apoB and cysteine 4057 in the kringle IV type 9 region of apo(a). Apo(a) is highly glycosylated and variable in length depending on the number of repeats of kringle IV type 2, which range from 3 to >40.^2^ There is an inverse relationship between the number of kringle IV type 2 repeats and Lp(a) concentration in white populations, but the degree of the inverse association varies among different racial/ethnic groups.^2,3^ About 80% of whites have Lp(a) levels <100 nmol/L, but because the distribution of Lp(a) concentrations is markedly skewed to the right, some individuals have plasma Lp(a) concentrations as high as 800 nmol/L.^3^ However, there is a marked difference in Lp(a) distribution among different racial groups with individuals of African descent having substantially higher Lp(a) levels than whites or other racial groups.^2^ A recent meta-analysis showed an increase in cardiovascular disease risk in individuals with Lp(a) levels >70 nmol/L.^4^ [Lp(a) is a protein with many isoforms and hence its molecular mass varies, making it difficult to use mg/dL as standard units of quantification.^5^ For results reported in nanomole per liter of apo(a), most investigators and clinicians rely on using the conversion of 2.4 to convert the results from nanomole per liter of apo(a) to the more familiar milligrams per deciliter of total Lp(a) particle. However, this assumes a constant mass of Lp(a) of 400 kDa, which is unlikely considering that apo(a) mass ranges from 300 to 800 kDa.]

Statins and ezetimibe are cardioprotective because of their ability to reduce plasma levels of the major atherogenic apoB lipoproteins.^6,7^ They do not, however, reduce levels of Lp(a), a factor that contributes to residual cardiovascular risk.^8^ In contrast, the recently approved PCSK9 inhibitors do lower plasma Lp(a) levels^9,10^ as does anacetrapib, one of the remaining CETP (cholesteryl ester transfer protein) inhibitors in development.^11^

Anacetrapib, a small-molecule inhibitor of CETP, is associated with a dose-dependent decrease in plasma Lp(a) when given as a monotherapy.^12^ In the DEFINE trial (Determining the Efficacy and Tolerability of CETP Inhibition With Anacetrapib), a study of ≈ 1600 subjects, 100 mg/d of anacetrapib given with a statin was associated with a 23.8% reduction in Lp(a) after 24 weeks and 17.1% after 76 weeks. Early kinetic studies suggested that baseline levels of Lp(a) are primarily determined by production rather than fractional clearance.^13,14^ However, we recently reported that the PCSK9 inhibitor, alirocumab, reduced Lp(a) concentrations by increasing the clearance of apo(a) without affecting its production.^10^ To understand the mechanism by which anacetrapib lowers Lp(a), we examined apo(a) metabolism using stable isotopes and novel liquid chromatography–mass spectrometry (LC/MS) methods.^10,15^

## Materials and Methods

Materials and Methods are available in the online-only Data Supplement.

## Results

In this exploratory study, we performed stable isotope studies of apo(a) kinetics in 12 subjects chosen from a larger cohort of 39 individuals who had participated in studies of the effects of anacetrapib on the metabolism of VLDL (very-low–density lipoprotein), IDL (intermediate-density lipoprotein), LDL, and HDL.^16–18^ The 12 subjects had Lp(a) levels ≥20 nmol/L at the end of the first treatment period (baseline) and at least a 15% reduction in Lp(a) while receiving anacetrapib. The basis for the inclusion criterion of 20 nmol/L of Lp(a) at the end of the placebo/atorvastatin period derived from limitations in the sensitivity of the LC-MS/MS method. In the absence of any data on intraindividual variability of the effects of CETP inhibition on Lp(a) clearance, we chose the criteria of a 15% reduction in Lp(a) levels during anacetrapib treatment. We excluded 1 subject (subject 9 in Table I in the online-only Data Supplement) who met the criteria for Lp(a) levels after the first treatment period but whose post-treatment Lp(a) level on anacetrapib was below the sensitivity of the LC/MS method. We were also unable to carry out studies in 2 other subjects who met inclusion criteria (subject 14 and subject 31) because of inadequate samples left after completion of our primary studies of LDL and HDL metabolism.^16–18^

Table 1 lists the demographic characteristics and baseline lipid values. Baseline values were obtained at the end of the placebo/statin run-in period. There were 7 women and 5 men, and 8 of the 12 subjects identified themselves as black (66.7%). Mean age was 49.2 years, and mean body mass index was 30.6 kg/m^2^. Plasma LDL-C levels were 134.9 mg/dL. There were no significant differences in baseline characteristics between the overall cohort of 39 subjects^16^ and the 12 individuals chosen to participate in the present study.

**Table 1.**
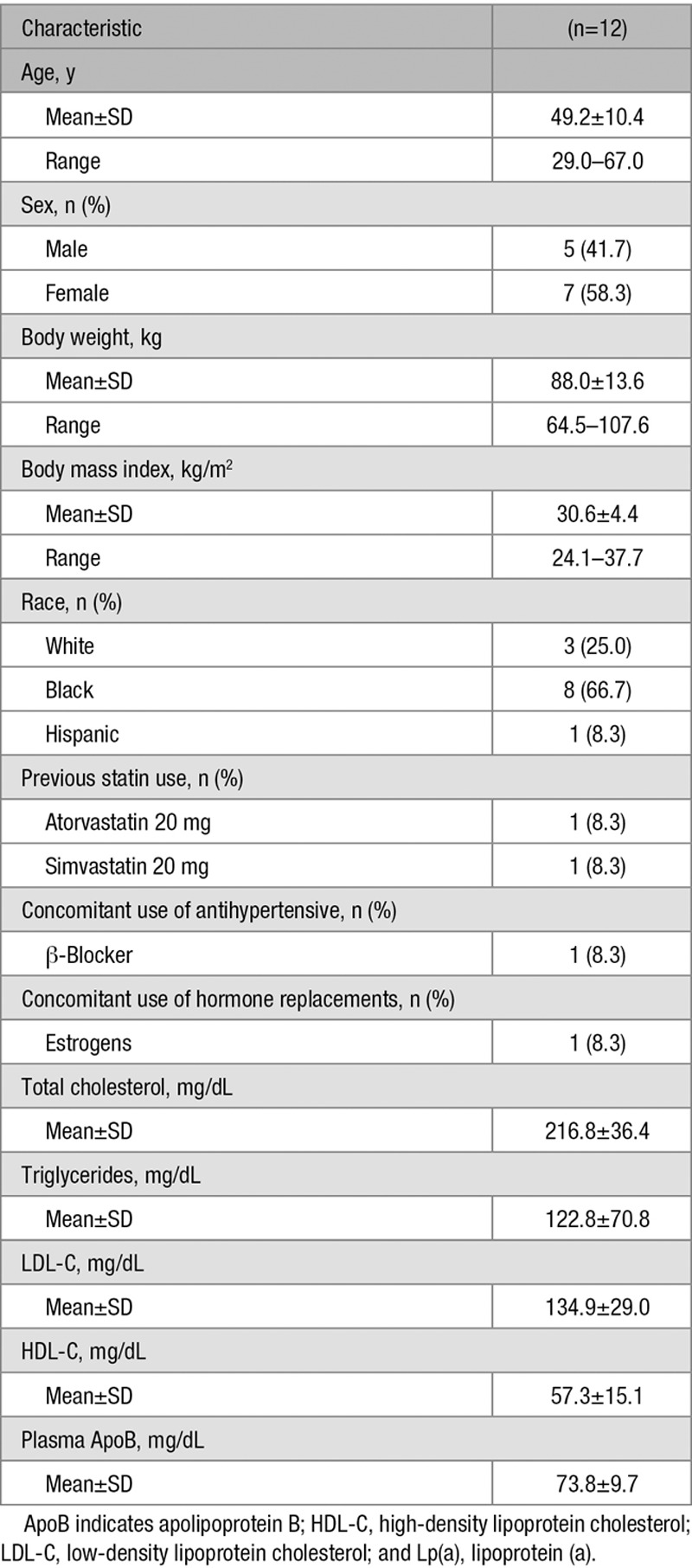
Demographic Characteristics and Baseline Lipids of Subjects With Lp(a) Kinetic Studies

### Plasma Levels of Lp(a) and Isoform Size

The median and interquartile range of plasma Lp(a) levels for the complete cohort was 21.5 nmol/L (9.9–108.1 nmol/L) after the first treatment period and 15.8 nmol/L (5.2–83.4 nmol/L) after anacetrapib treatment. Isoform sizes ranged from 13 to 32 repeats, with the distribution skewed toward larger size isoforms (Table I in the online-only Data Supplement). Lp(a) levels in the entire cohort were lowered by 34.1%. The Lp(a) percent decrease was similar for the statin–anacetrapib group (34.5%) and placebo–anacetrapib group (32.6%; Table 2). The median and interquartile range of plasma Lp(a) levels for the cohort of 12 subjects undergoing apo(a) kinetics studies were 52.9 nmol/L (38.4–121.3 nmol/L) after placebo or statin alone and 34.3 nmol/L (16.1–85.2 nmol/L) after 8 weeks of anacetrapib treatment. This decrease in Lp(a) levels of 39.6% was highly significant (*P*≤0.001). The decreases in levels were similar for the statin–anacetrapib group (34.5%) and placebo–anacetrapib group (32.6%; Table 2). The similar reductions in plasma Lp(a) levels within our subgroup (median Lp(a) of 52.9 nmol/L) and the overall cohort (median Lp(a) 21.5 nmol/L) suggest that anacetrapib-mediated reductions in Lp(a) may be independent of baseline levels.

**Table 2.**
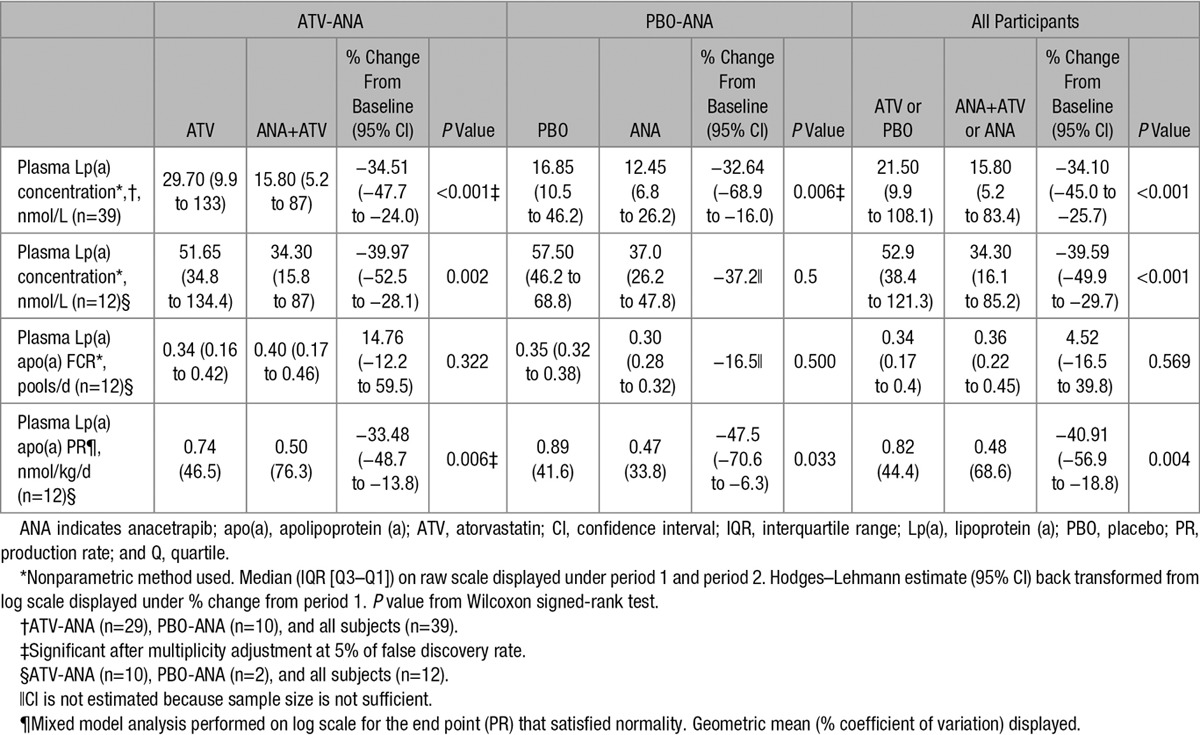
Effect of Anacetrapib on Lp(a) Plasma Levels, Fractional Clearance Rates, and PRs

### Apo(a) Metabolism

The apo(a) fractional catabolic rate (FCR; median and interquartile range) was 0.34 pools/d (0.17–0.41 pools/d) at the end of the first treatment period and 0.36 pools/d (0.21–0.45 pools/d) at the end of anacetrapib treatment. The difference of 4.5% (95% confidence interval, −16.5% to 39.8%) was not significant (*P*=0.57; Table 2). Apo(a) FCRs at baseline ranged from 0.09 to 0.41 pools/d in the 12 subjects studied (Table II in the online-only Data Supplement). The FCRs at the end of period 1 were similar between the group on anacetrapib plus atorvastatin (n=10) and the anacetrapib-only group (n=2; Table 2), and there were no effects of anacetrapib on apo(a) FCR in either group alone or when the data were combined. The absence of an effect of anacetrapib on FCR suggest that the reductions observed in Lp(a) plasma levels after treatment were because of a change in apo(a) production rate (PR).

The apo(a) PR (geometric mean and % coefficient of variation) was 0.82 nmols/kg/d (44.4 nmols/kg/d) at the end of period 1 and 0.48 nmols/kg/d (68.6 nmols/kg/d) after 8 weeks of anacetrapib treatment. Treatment with anacetrapib for 8 weeks reduced geometric mean Lp(a) PR by 40.9% (95% confidence interval, −57.0% to −18.9%; *P*=0.004; Table 2). Lp(a) PR was reduced in 10 of the 12 subjects. The 2 subjects who did not have reductions in their PRs (subjects 2 and 5; Table II in the online-only Data Supplement) had large reductions in Lp(a) levels, from 199.70 to 123.40 nmol/L and 164.40 to 83.40 nmol/L, respectively. These changes were associated with large increases in FCRs. These subjects were in the statin–anacetrapib arm of the study.

## Discussion

Increasing evidence supporting a role of Lp(a) in the pathogenesis of cardiovascular disease, together with the development of several novel therapeutics that significantly reduce plasma levels of both LDL and Lp(a), make it imperative to extend our understanding of the metabolism of Lp(a).^19^ The current study examined the effects of CETP inhibition on the clearance and production of Lp(a). Our study found that decreases in apo(a) production are responsible for lowering of Lp(a) after anacetrapib treatment. Apo(a) is synthesized by the liver, and studies in cells, animal models, and humans have provided evidence that Lp(a) is either assembled from apoB and apo(a) within or at the hepatocyte surface, or assembled from apoB-containing lipoproteins and apo(a) in the circulation.^20–23^ Much work needs to be done to determine whether either of these pathways or a combination of pathways, depending on differing metabolism states, are driving Lp(a) assembly. Regardless of where Lp(a) is assembled, most studies have supported the PR of Lp(a) as regulating its plasma levels.^13,24^ However, when the basis of pharmacological changes in Lp(a) levels has been interrogated, a mixed picture has emerged. Thus, whereas some investigators observed a reduction in Lp(a) PR, such as after estrogen- or niacin-induced decreases in plasma Lp(a) concentrations,^20,25,26^ our recent study of the effects of inhibition of PCSK9 with alirocumab on Lp(a) metabolism suggested that the reduction in Lp(a) concentration was because of an increase in the FCR of Lp(a), without any change in PR.^17^ The demonstration of reduced apo(a) PR as the basis for anacetrapib-mediated reductions in Lp(a) levels must be viewed in the context of the lack of a full understanding of how this lipoprotein is produced. Although apo(a)/Lp(a) secretion is regulated nearly completely at the *LPA* locus through both the number of repeats of kringle IV type 2 and additional sequence variation unrelated to kringles,^27,28^ numerous response elements for transcription factors and nuclear receptors have been identified in the promoter of the *LPA* gene, including a response element for FXR (farnesoid X receptor), a nuclear receptor that plays a key role in hepatic cholesterol metabolism.^28^ Post-transcriptional modulation of apo(a) secretion has been suggested by studies of Nassir et al,^29^ who showed that oleate increased, and MTP (microsomal triglyceride transfer protein) inhibition decreased, the secretion of an apo(a) peptide from HepG2 cells. Finally, a recent publication by Sharma et al^30^ demonstrated recycling of apo(a) after uptake of Lp(a) by hepatocytes, allowing for expansion of the concept of post-transcriptional regulation of Lp(a) production to include Lp(a)–apo(a) recycling as a component of measured Lp(a) production.

CETP inhibition does alter VLDL core lipid composition, with triglyceride (TG) enrichment resulting from the lack of exchange with HDL cholesteryl ester (CE), and TG-enriched VLDL (often referred to as VLDL1) may be removed directly by the liver more than normal TG.^31^ If apo(a) at the surface of the liver binds to a TG-rich lipoprotein such as newly secreted VLDL that, as a result of CETP inhibition, is removed by the liver directly without conversion to more dense lipoproteins, which could result in a fall in the PR of the mature Lp(a) that we isolated at density: 1.019 to 1.210 g/mL. Although we previously reported that anacetrapib treatment increased the FCRs of both VLDL and IDL apoB, the conversion of VLDL to LDL was ≈90% during both placebo and anacetrapib treatment periods.^16^ Thus, it is unlikely that greater hepatic clearance of a TG-rich Lp(a) precursor during anacetrapib administration accounted for the reduction in Lp(a) PR that we observed. In addition, we determined the FCR of apo(a) in the VLDL/IDL fraction in our subjects, and it was similar to the FCR of apo(a) in the LDL/HDL (data not shown). Because 90% of Lp(a) is in the LDL/HDL density range, a small VLDL/IDL Lp(a) pool with an FCR similar to that of Lp(a) in the much larger LDL/HDL density range could not be a significant precursor to the latter. At present, we do not have a clear explanation for the reduction in Lp(a) PR that we observed during inhibition of CETP activity with anacetrapib. Additional studies in cells or rodent models that have been modified to produce Lp(a) should be conducted to examine the mechanisms whereby CETP inhibition, estrogen treatment, and niacin therapy all reduce Lp(a) production.

The absence of any change in the FCR of Lp(a) is noteworthy because, in the overall cohort, we found that anacetrapib treatment was associated with a significant 18% decrease in LDL apoB concentration because of an 18% increase in the FCR of LDL apoB without any effect on PR.^16^ We were unable to identify the basis of the increased FCR for LDL apoB with anacetrapib treatment, although an increase in the affinity of LDL for its receptor or increased numbers of LDL receptors seems most likely. Supporting this possibility is work demonstrating that overexpression of CETP in mice is associated with decreased hepatic LDL receptor gene expression^32^; inhibition of CETP might, therefore, increase LDL receptors. If that was the case, it would be more evidence that Lp(a) and apo(a) are not removed from the circulation via LDL receptors in the liver. This would be consistent with the absence of effects of either statins or ezetimibe on Lp(a) concentrations. Our recent study of the effect of alirocumab on apoB and Lp(a) metabolism suggest, however, that the much larger increases in LDL receptor number during PCSK9 treatment versus statin or ezetimibe treatment, as indicated by the greater LDL-lowering capacities of PCSK9 inhibitors, may be needed to see increased clearance of Lp(a) via the LDL receptor pathway. We cannot rule out that changes in the core lipid composition of the Lp(a) particle during CETP treatment might reduce the affinity of Lp(a) for LDL receptors, negating any increase in the number of receptors resulting from inhibition of CETP activity.

Our study results may have been affected by studying a cohort with blacks as a majority. It is accepted that Lp(a) levels are affected by race, and the basis for this difference is not clear. We could not examine the effect of race in this subpopulation of our study. We acknowledge, therefore, that our results cannot be generalized to a broader population.

In conclusion, reductions of Lp(a) of ≥15% in mildly hypercholesterolemic subjects treated with the CETP inhibitor anacetrapib were because of reductions in apo(a) PR with no changes in apo(a) FCR. The underlying mechanism responsible for the decrease in Lp(a) production is unclear and will require additional work in preclinical models.

## Acknowledgments

Editorial assistance was provided by Jennifer Rotonda of Merck & Co, Inc, Kenilworth, NJ, and laboratory assistance was provided by Colleen Ngai of Columbia University, New York, NY. T. Thomas, H. Zhou, W. Karmally, R. Ramakrishnan, S. Holleran, Y. Liu, P. Jumes, J.A. Wagner, B. Hubbard, S.F. Previs, T. Roddy, A.O. Johnson-Levonas, D.E. Gutstein, S.M. Marcovina, D.J. Rader, H.N. Ginsberg, J.S. Millar, and G. Reyes-Soffer are responsible for the work described in this article. All authors were involved in at least one of the following: conception, design, acquisition, analysis, statistical analysis, and interpretation of data in addition to drafting the article and revising/reviewing the article for important intellectual content. All authors provided final approval of the version to be published and agree to be accountable for all aspects of the work in ensuring that questions related to the accuracy or integrity of any part of the work are appropriately investigated and resolved.

## Sources of Funding

Merck & Co, Inc, Kenilworth, NJ, provided financial support for the conduct of the study.

## Disclosures

H. Zhou, Y. Liu, P. Jumes, J.A. Wagner, B. Hubbard, S.F. Previs, T. Roddy, A.O. Johnson-Levonas, and D.E. Gutstein are or were employees of Merck Sharp & Dohme Corp, a subsidiary of Merck & Co, Inc, Kenilworth, NJ, and may own stock and hold stock options in the Company. J.S. Millar and G. Reyes-Soffer received grant support and honoraria from Merck and consulting fees outside of the submitted work. H.N. Ginsberg reports grants and consulting fees from Merck during the conduct of the study and consulting fees from Amgen, Kowa, Sanofi, Regeneron, Lilly, Boerhinger Ingelheim, and Pfizer outside the submitted work. S.M. Marcovina reports consulting fees from Denka Seiken, Ltd, and MedTest Dx outside the submitted work. D.J. Rader is a member of the Merck Scientific Advisory Board. The other authors declare no conflicts.

## Supplementary Material
