# Supplementary material for: CETP (Cholesteryl Ester Transfer Protein) Inhibition With Anacetrapib Decreases Production of Lipoprotein(a) in Mildly Hypercholesterolemic Subjects
Source: Arterioscler Thromb Vasc Biol. 2017 Aug 23;37(9):1770–5. doi: 10.1161/ATVBAHA.117.309549 (PMC5567403; doi:10.1161/ATVBAHA.117.309549)
Supplement: Supplementary file 1 [file atv-37-1770-s001.pdf]

MATERIALS AND METHODS

Study Population

This study (Merck Protocol number 0859-026-03; NCT00990808) was conducted at the Columbia University Medical Center and the University of Pennsylvania between December 2009 and July 2011. The study was approved by the Human Investigation Review Board of each institution and all participants gave informed consent before enrollment into the study. The primary goal of the overall study was to determine how CETP inhibition altered apoB and apoAI metabolism. To achieve that goal, 39 participants were enrolled in a fixed-sequence, single-blind study of the effects of anacetrapib on the metabolism of VLDL, IDL, LDL, and HDL. Twenty-nine were randomized to atorvastatin 20mg/day, plus placebo for 4 weeks (period 1), and then atorvastatin plus anacetrapib (100 mg/day) for 8 weeks (period 2). The other 10 subjects were randomized to double placebo for 4 weeks followed by placebo plus anacetrapib for 8 weeks.[1,2] The goal of the current sub-study was to determine the mechanism by which CETP inhibition reduces Lp(a). Hence, only subjects with Lp(a) reductions greater than 20% after 8 weeks of anacetrapib treatment were included. In addition, we only studied subjectswith baseline Lp(a) concentrations greater than 15nmol/L (a concentration that allowed us to reliably determine apo(a) enrichment by our LC/MS method).[3] The study design included two arms with two periods within each arm.[1,2] Ten of the 12 subjects studied were in the group that received atorvastatin, 20mg/day, plus placebo for 4 weeks followed by atorvastatin plus anacetrapib (100 mg/day) for 8 weeks. The remaining 2 subjects were in the group that received double placebo for 4 weeks followed by placebo plus anacetrapib for 8 weeks. These two subjects had baseline characteristics and Lp(a) levels similar to the other 10 participants

and, therefore, we combined the groups for data analysis. We analyzed apo(a) flux in a 1:1 (vol:vol) combination of LDL (d:1.019-1.063 g/mL) and HDL (d:1.063-1.21 g/mL) fractions isolated by ultracentrifugation.[3] About 90 to 95% of the total plasma Lp(a) particles fall within the LDL and HDL density range.[4]

Research Protocol

The study protocol has been previously described.[1] Briefly, isocaloric low-fat liquid meals (57% carbohydrate, 18% fat, 25% protein) were started 8 hours before the administration of stable isotopes (1 am on Day1) and provided to subjects every 2 hrs for the next 32 hrs to maintain a steady state during the kinetic study. For the analysis of apo(a), we utilized only one of the three stable isotopes administered during the parent study[1] at 9am on day 2, subjects received a bolus injection of [5,5,5,-$^{2}H_{3}$](D3)-leucine (10 umol/kg body weight) immediately followed by a constant infusion of D3-leucine (10 umol/kg body weight/hour) for 15 hours. Blood samples collected at 0, 2, 4, 6, 8, 10, 12, 15 hours and stored at -80 degrees were utilized for this sub-study examining the kinetics of Lp(a). The combined d:1019-1.21 g/ml (LDL+HDL) fractions were desalted, reduced with DTT, alkylated with iodoacetamide, and digested with trypsin.[3]

LC-MS/MS Analysis

A peptide specific to apo(a) was identified and used for the kinetic analysis: LFLEPTQADIALLK. The digested samples were injected onto a Waters Trizaic Nanotile microfluidic device (150 μm × 100 mm packed with BEH C18, 1.7-μm particle) using a NanoAcquity UPLC system and were analyzed with a Xevo TQ-S tandem quadrupole mass spectrometer (Waters Corporation) in multiple reaction mode (MRM). Transitions

were monitored for the unlabeled (786.5→1069.5) and labeled peptides (788→1069.5). The transition used for monitoring the labeled peptide can detect the incorporation of a labeled leucine into the first or third amino acid in the LFLEPTQADIALLK peptide therefor the enrichment was divided by 2 before the calculation of apo(a) FCR.

Kinetic Calculations

The FCR was calculated by the linear change in apo(a) enrichment between 4 to 15 hrs relative to the intracellular precursor enrichment, assumed to be the asymptotic enrichment of leucine in VLDL apoB.[3] Apo(a) PR was calculated as the product of the FCR and the molar pool size of apo(a), which is the apo(a) concentration (nM) multiplied by the plasma volume (taken as 0.045 L/kg body weight).

Lp(a) Plasma Measurements

Plasma Lp(a) levels were measured by a double monoclonal antibody-based ELISA that has been demonstrated to be unaffected by the variable number of kringle IV type 2 repeats in the patient samples.[5] Apo(a) isoforms were determined by a high sensitive agarose gel electrophoresis method followed by immunoblotting as previously described,[6] in which it has been demonstrated that there is a log correlation between the mobility of the isoform on the gel and the number of Kringle IV type 2 repeats. In very few subjects, only 1 apo(a) isoform is expressed. In most individuals, however, both apo(a) alleles are expressed, with 1 isoform usually, more prevalent than the other. Particle sizes and number were determined by ion mobility analysis.[3] These were

measured in the complete cohort of thirty-nine participants and are presented in Supplemental Table I.

Statistics

Analysis was performed on log-scale and the estimates obtained were back-transformed using the formula 100*(exp (estimate)-1) to yield point estimates, 95% confidence intervals, and between-treatment p-values (two tailed) for the true percent change from period 1. Depending on the distribution of the data, different analytic approaches were used. For normally distributed data, linear mixed effects models containing fixed effects for the two study arms and treatment within each arm, and random effect for subject within panel were used. For non-normally distributed data, Hodges-Lehmann estimates based on Wilcoxon signed rank test and corresponding p-values were reported for treatment period differences (Period 2 – Period 1), as well as median and inter-quartile range for individual treatment periods. A two-stage false discovery rate (FDR) method was used to control the FDR for the comparisons of anacetrapib with atorvastatin versus anacetrapib alone, and anacetrapib versus placebo at 5% for Lp(a) concentration, Lp(a) FCR, Lp(a) PS, Lp(a) PR.

References:

1. Millar JS, Reyes-Soffer G, Jumes P et al. Anacetrapib lowers LDL by increasing ApoB clearance in mildly hypercholesterolemic subjects. *J Clin Invest* 2015;125:2510-2522.
2. Reyes-Soffer G, Millar JS, Ngai C et al. Cholesteryl Ester Transfer Protein Inhibition With Anacetrapib Decreases Fractional Clearance Rates of High-Density Lipoprotein Apolipoprotein A-I and Plasma Cholesteryl Ester Transfer Protein. *Artheroscler Thromb Vasc Biol* 2016;36:994-1002.
3. Zhou H, Castro-Perez J, Lassman ME. Measurement of apo(a) kinetics in human subjects using a microfluidic device with tandem mass spectrometry. *Rapid Commun Mass Spectrom* 2013;27:1294-1302.
4. Gries A, Nimpf J, Nimpf M, Wurm H, Kostner GM. Free and Apo B-associated Lpa-specific protein in human serum. *Clin Chim Acta* 1987;164:93-100.
5. Marcovina SM, Albers JJ, Gabel B, Koschinsky ML, Gaur VP. Effect of the number of apolipoprotein(a) kringle 4 domains on immunochemical measurements of Lipopotein(a). *Clin Chem* 1995;41:246-255.
6. Marcovina SM, Zhang ZH, Gaur VP, Albers JJ. Identification of 34 apolipoprotein(a) isoforms: Differential expression of apolipoprotein(a) alleles between American Blacks and Whites. *Biochem Biophys Res Commun* 1993;191:1192-1196.
