# Supplementary material for: CETP (Cholesteryl Ester Transfer Protein) Inhibition With Anacetrapib Decreases Production of Lipoprotein(a) in Mildly Hypercholesterolemic Subjects
Source: Arterioscler Thromb Vasc Biol. 2017 Aug 23;37(9):1770–5. doi: 10.1161/ATVBAHA.117.309549 (PMC5567403; doi:10.1161/ATVBAHA.117.309549)
Supplement: Supplementary file 2 [file atv-37-1770-s002.pdf]

**Supplemental Table I.** Full cohort Lp(a) levels and isoform size (N=39)

| Study Arm | Subject Number | Plasma Lp(a) levels (nmol/L) Period 1 | Period 2 | apo(a) isoforms (number of Kringle 4 domains) | | |
|---|---|---|---|---|---|---|
| S-ANA | 1 | 134.4 | 96.7 | 19 | / | 20 |
| S-ANA | 2 | 199.7 | 123.4 | 18 | / | 25 |
| S-ANA | 3 | 42.0 | 13.2 | 28 | / | 31 |
| S-ANA | 4 | 211.5 | 205.9 | 17 | / | 31 |
| S-ANA | 5 | 164.4 | 83.4 | 24 | / | 27 |
| S-ANA | 6 | 34.8 | 15.8 | 26 | / | 30 |
| S-ANA | 7 | 0.2 | 0.2 | 28 | / | - |
| S-ANA | 8 | 236.3 | 223.8 | 17 | / | 23 |
| S-ANA | 9** | 20.6 | 6.7 | 23 | / | 25 |
| S-ANA | 10 | 20.4 | 14.0 | 30 | / | 35 |
| S-ANA | 11 | 277.0 | 265.0 | 14 | / | 19 |
| S-ANA | 12 | 29.7 | 16.4 | 21 | / | 32 |
| S-ANA | 13 | 59.6 | 31.3 | 20 | / | 22 |
| S-ANA | 14* | 21.5 | 16.7 | 28 | / | - |
| S-ANA | 15 | 133.0 | 128.0 | 22 | / | 19 |
| S-ANA | 16 | 1.4 | 0.2 | 32 | / | 28 |
| S-ANA | 17 | 5.3 | 2.7 | 32 | / | 34 |
| S-ANA | 18 | 17.9 | 13.3 | 25 | / | 36 |
| S-ANA | 19 | 30.4 | 28.9 | 28 | / | 19 |
| S-ANA | 20 | 7.0 | 5.2 | 20 | / | 23 |
| S-ANA | 21 | 1.3 | 0.7 | 31 | / | 33 |
| S-ANA | 22 | 15.2 | 3.7 | 23 | / | 27 |
| S-ANA | 23 | 43.7 | 37.3 | 25 | / | 29 |
| S-ANA | 24 | 5.2 | 4.3 | 28 | / | 30 |
| S-ANA | 25 | 108.1 | 87.0 | 18 | / | 28 |
| S-ANA | 26 | 209.3 | 180.6 | 16 | / | 25 |
| S-ANA | 27 | 1.0 | 0.3 | 26 | / | 31 |
| S-ANA | 28 | 9.9 | 9.1 | 18 | / | 23 |
| S-ANA | 29 | 17.2 | 9.0 | 19 | / | 26 |
| P-ANA | 30 | 13.6 | 8.8 | 28 | / | 34 |
| P-ANA | 31* | 22.2 | 16.1 | 13 | / | 28 |
| P-ANA | 32 | 1.2 | 0.7 | 32 | / | 34 |
| P-ANA | 33 | 68.8 | 47.8 | 23 | / | 28 |
| P-ANA | 34 | 46.2 | 26.2 | 29 | / | 26 |
| P-ANA | 35 | 0.9 | 0.1 | 28 | / | 30 |
| P-ANA | 36 | 20.1 | 24.8 | 27 | / | 29 |
| P-ANA | 37 | 10.5 | 8.4 | 19 | / | 28 |
| P-ANA | 38 | 11.9 | 6.8 | 18 | / | 31 |
| P-ANA | 39 | 123.4 | 106.9 | - | / | - |

S-ANA: Atorvastatin-Anacetrapib Arm, P-ANA: Placebo-Anacetrapib Arm
*Met inclusion criteria but did not have adequate sample volumes remaining after completion of prior (main) studies.
**Met baseline inclusion criteria but post treatment Lp(a) levels were below the sensitivity of the LC/MS method.

**Supplemental Table II.** Individual Subject apo(a) concentrations, fractional clearance rates and production rates.

| | Subject Number | Pool Size apo(a) nM Period 1 | Pool Size apo(a) nM Period 2 | FCR pools/d Period 1 | FCR pools/d Period 2 | PR nmol/kg/d Period 1 | PR nmol/kg/d Period 2 | % Δ apo(a) | % Δ FCR | % Δ PR |
|---|---|---|---|---|---|---|---|---|---|---|
| S-ANA | 1 | 134.40 | 96.70 | 0.179 | 0.159 | 1.084 | 0.691 | -28% | -11% | -36% |
| S-ANA | 2* | 199.70 | 123.40 | 0.155 | 0.253 | 1.397 | 1.403 | -38% | 63% | 0% |
| S-ANA | 3 | 42.00 | 13.20 | 0.344 | 0.386 | 0.650 | 0.229 | -69% | 12% | -65% |
| S-ANA | 5* | 164.40 | 83.40 | 0.143 | 0.372 | 1.054 | 1.398 | -49% | 161% | 33% |
| S-ANA | 6 | 34.80 | 15.80 | 0.316 | 0.475 | 0.495 | 0.338 | -55% | 50% | -32% |
| S-ANA | 10 | 20.40 | 14.00 | 0.413 | 0.459 | 0.380 | 0.289 | -31% | 11% | -24% |
| S-ANA | 12 | 29.70 | 16.40 | 0.385 | 0.378 | 0.514 | 0.279 | -45% | -2% | -46% |
| S-ANA | 13 | 59.60 | 31.30 | 0.389 | 0.417 | 1.042 | 0.588 | -47% | 7% | -44% |
| S-ANA | 23 | 43.70 | 37.30 | 0.299 | 0.159 | 0.588 | 0.267 | -15% | -47% | -55% |
| S-ANA | 25 | 108.10 | 87.00 | 0.086 | 0.075 | 0.420 | 0.293 | -20% | -13% | -30% |
| P-ANA | 33 | 68.80 | 47.80 | 0.346 | 0.259 | 1.072 | 0.557 | -31% | -25% | -48% |
| P-ANA | 34 | 46.20 | 26.20 | 0.305 | 0.294 | 0.634 | 0.346 | -43% | -4% | -45% |

*Data from the two subjects who had no change or increase in PR.
