# Supplementary material for: CETP (Cholesteryl Ester Transfer Protein) Inhibition With Anacetrapib Decreases Production of Lipoprotein(a) in Mildly Hypercholesterolemic Subjects
Source: Arterioscler Thromb Vasc Biol. 2017 Aug 23;37(9):1770–5. doi: 10.1161/ATVBAHA.117.309549 (PMC5567403; doi:10.1161/ATVBAHA.117.309549)
Supplement: Supplementary file 3 [file atv-37-1770-s003.pdf]

Potential mechanisms for decreased Lp(a) production during inhibition of CETP

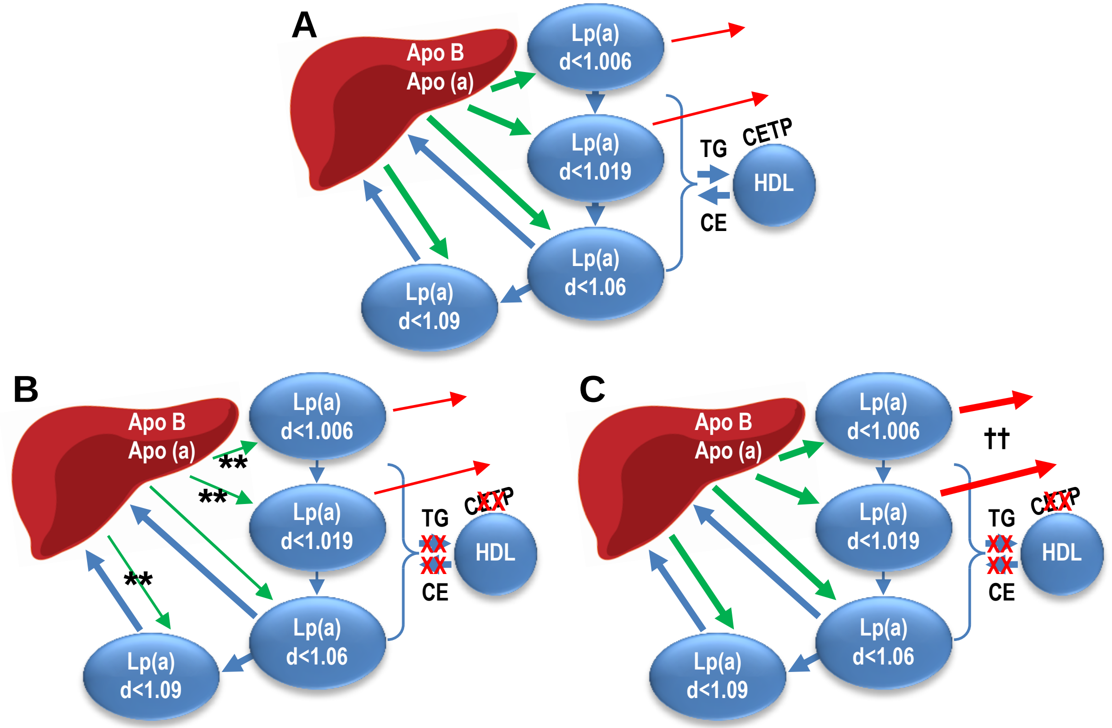

In the untreated state with normal CETP activity **(A),** apoB and apo(a) merge within or at the surface of the liver with the possibility that the resultant Lp(a) has a density like VLDL (D<1.006), IDL (d<1.019), LDL (D<1.06), and/or HDL (d<1.09). With lipolysis and remodeling of apoB particles by CETP, Lp(a) matures to its predominant densities in the LDL and HDL range. There may also be some direct removal of larger Lp(a) particles before maturation of Lp(a) is complete. Finally, mature Lp(a) (LDL and HDL density size) is cleared by the liver. Several variations of this model have been proposed by other investigators. We have chosen to present what we believe is the simplest model to explain the majority of published data. In this study, we observed that when CETP was inhibited by anacetrapib, levels of Lp(a) fell due to reductions of mature Lp(a) production in the LDL/HDL density range. This could be due to reduced hepatic secretion of Lp(a)** **(B),** or increased direct removal of larger Lp(a) particles†† **(C).** At present, we cannot distinguish between these two possibilities.
